# Genomic analysis of two Chinese isolates of hyphantria cunea nucleopolyhedrovirus reveals a novel species of alphabaculovirus that infects hyphantria cunea drury (lepidoptera: arctiidae)

**DOI:** 10.1186/s12864-022-08604-7

**Published:** 2022-05-13

**Authors:** Xiaowei Peng, Wenying Zhang, Chengfeng Lei, Shuifa Min, Jia Hu, Qinghua Wang, Xiulian Sun

**Affiliations:** 1 Wuhan Institute of Virology, Chinese Academy of Sciences, Wuhan, 430071 Hubei China; 2 University of Chinese Academy of Sciences, Beijing, 100049 China; 3 Hubei Ecology Polytechnic College, Wuhan, 430200 Hubei China; 4 Institute of Forestry Ecology, Environment and Nature Conservation, Chinese Academy of Forestry, Haidian, Beijing, 100091 China

**Keywords:** HycuNPV, Chinese isolates, SNPs, Baculovirus, Genomics, Diversity

## Abstract

**Background:**

Baculoviruses act as effective biological control agents against the invasive pest *Hyphantria cunea* Drury. In this study, two Chinese *Hyphantria cunea* nucleopolyhedrovirus (HycuNPV) isolates, HycuNPV-BJ and HycuNPV-HB, were deep sequenced and compared with the Japanese isolate, HycuNPV-N9, to determine whole-genome level diversity and evolutionary history.

**Results:**

The divergence of the phylogenetic tree and the K2P distances based on 38 core-gene concatenated alignment revealed that two Chinese HycuNPV isolates were a novel species of *Alphabaculovirus* that infected *Hyphantria cunea* in China. The gene contents indicated significant differences in the HycuNPV genomes between the Chinese and Japanese isolates. The differences included gene deletions, acquisitions and structural transversions, but the main difference was the high number of single nucleotide polymorphisms (SNPs). In total, 10,393 SNPs, corresponding to approximately 8% of the entire HycuNPV-N9 genome sequence, were detected in the aligned reads. By analyzing non-synonymous variants, we found that hotspot mutation-containing genes had mainly unknown functions and most were early expressing genes. We found that the *hycu78* gene which had early and late promoter was under positive selection. Biological activity assays revealed that the infectivity of HycuNPV-HB was greater than that of HycuNPV-BJ, and the killing speed of HycuNPV-HB was faster than that of HycuNPV-BJ. A comparison of molecular genetic characteristics indicated that the virulence differences between the two isolates were affected by SNP and structural variants, especially the homologous repeat regions.

**Conclusions:**

The genomes of the two Chinese HycuNPV isolates were characterized, they belonged to a novel species of *Alphabaculovirus* that infected *Hyphantria cunea* in China. We inferred that the loss or gain of genetic material in the HycuNPV-HB and HycuNPV-BJ genomes resulted in new important adaptive capabilities to the *H. cunea* host. These results extend the current understanding of the genetic diversity of HycuNPV and will be useful for improving the applicability of this virus as a biological control agent.

**Supplementary Information:**

The online version contains supplementary material available at 10.1186/s12864-022-08604-7.

## Background

The fall webworm, *Hyphantria cunea* Drury (Lepidoptera: Arctiidae), is a worldwide quarantine pest and a highly invasive pest in China. It has caused significant damage to forests, fruit trees and ornamental trees [1, 2]. The pest is native to North America, ranging from Canada to Mexico, and it was first reported in China in 1979 [3]. It was first found in Liaoning Province in China and has spread widely, becoming established in 13 provinces, including 607 counties, over the last few years [4]. It reproduces rapidly, resulting in two to three generations per year in many provinces. Since the first discovery of *H. cunea* larvae in Hubei Province in 2016, it has been identified in 11 urban areas. The insect is polyphagous and feeds on fruit trees, crops and ornamental trees in China, especially cherry blossom, mulberry, ailanthus, ash, hawthorn, plum, peach, crabapple, poplar etc., it continues to spread southward, seriously threatening the ecological environment of the southern provinces of China.

Baculoviruses are insect-specific double-stranded DNA viruses that have narrow host ranges and high virulence levels, which makes them safe ecologically and environmentally friendly biopesticides [5]. The baculovirus *Hyphantria cunea* nucleopolyhedrovirus (HycuNPV) has been isolated from the larvae of the fall webworm [2, 6]. Restriction endonuclease analyses revealed that different isolates of HycuNPV show very high degrees of genotypic variation, which is reflected in the virulence against *H. cunea* larvae, as well as the yields of budded virions and polyhedra in cell cultures [7]. At present, several HycuNPV strains have been isolated in China and successfully used as biological insecticides against *H. cunea* [4, 8, 9]. However, to our knowledge, there are no reports comparing the molecular genetic characteristics of HycuNPV isolates at the whole-genomic level, and little is known about the genetic composition and the genomic diversity of the Chinese isolates.

In this study, the genomes of two Chinese HycuNPV isolates, HycuNPV-HB and HycuNPV-BJ, were first sequenced using a high-throughput method. We determined the genome sequences and organization of the two isolates and compared them with those of the first published HycuNPV genome sequence, the HycuNPV-N9 isolate from Japan (Accession number: NC_007767.1) [10]. We aimed to reveal the gene contents and organizational characteristics of the Chinese isolates, as well as the genetic diversity in HycuNPV populations. A detailed genetic comparison of HycuNPV will increase our understanding of the molecular mechanisms responsible for HycuNPV host-specificity and will be useful in developing efficient biological insecticides for the control of ***H. cunea*** populations in China.

## Results

### Electron microscopy

**Fig. 1:**
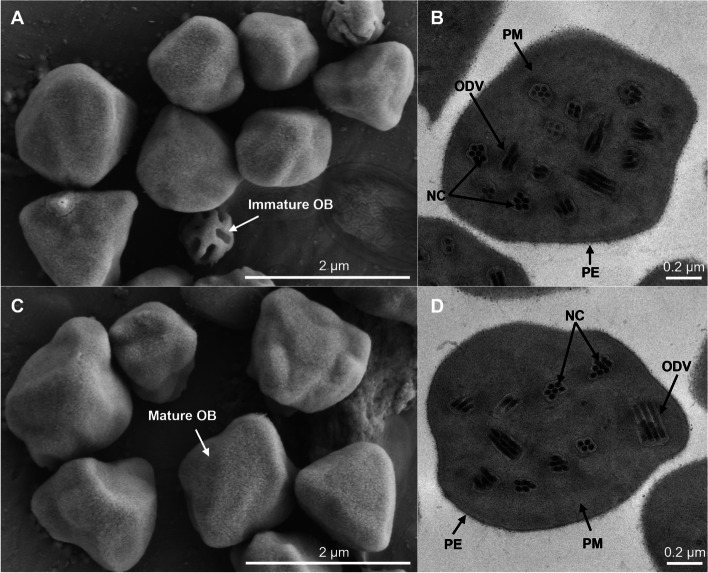
Image of scanning electron microscopy and transmission electron microscopy observation results for the occlusion bodies of HycuNPV-HB (**A, B**) and HycuNPV-BJ (**C, D**). **A, C** SEM (× 25,000) showing occlusion body shapes, the rod-shaped indentations on the surfaces of OBs reveals an immature OB. **B, D** TEM (× 14,500) showing the cross section of the occlusion bodies, ODV: occlusion-derived virus, NC: nucleocapsids, PE: polyhedrin envelop, PM: polyhedrin matrix

The two HycuNPV isolates were obtained from Hubei Province (HB) and Beijing (BJ) City, China, and were named as HycuNPV-HB and HycuNPV-BJ, respectively. Scanning Electron Microscopy (SEM) revealed that the purified occlusion bodies (OBs) of HycuNPV-HB are polyhedral or irregular in shape, the mean size of the pyramidal OBs was 1.3 ± 0.2 μm (Fig. 1A). Transmission Electron Microscopy (TEM) showed OBs containing many enveloped virions packaged in multiple nucleocapsids, the number ranged from 2 to 8, the length of a single viral nucleocapsid was 272 ± 10 nm and the diameter was 45 ± 15 nm (Fig. 1B). The morphology and sizes of OBs and virions in HycuNPV-BJ were similar to those in HycuNPV-HB (Fig. 1C and D). Electron micrographs of the Chinese HycuNPV isolates showed typical morphological characteristics observed in other alphabaculoviruses.

### Phylogenetic analysis

**Fig. 2:**
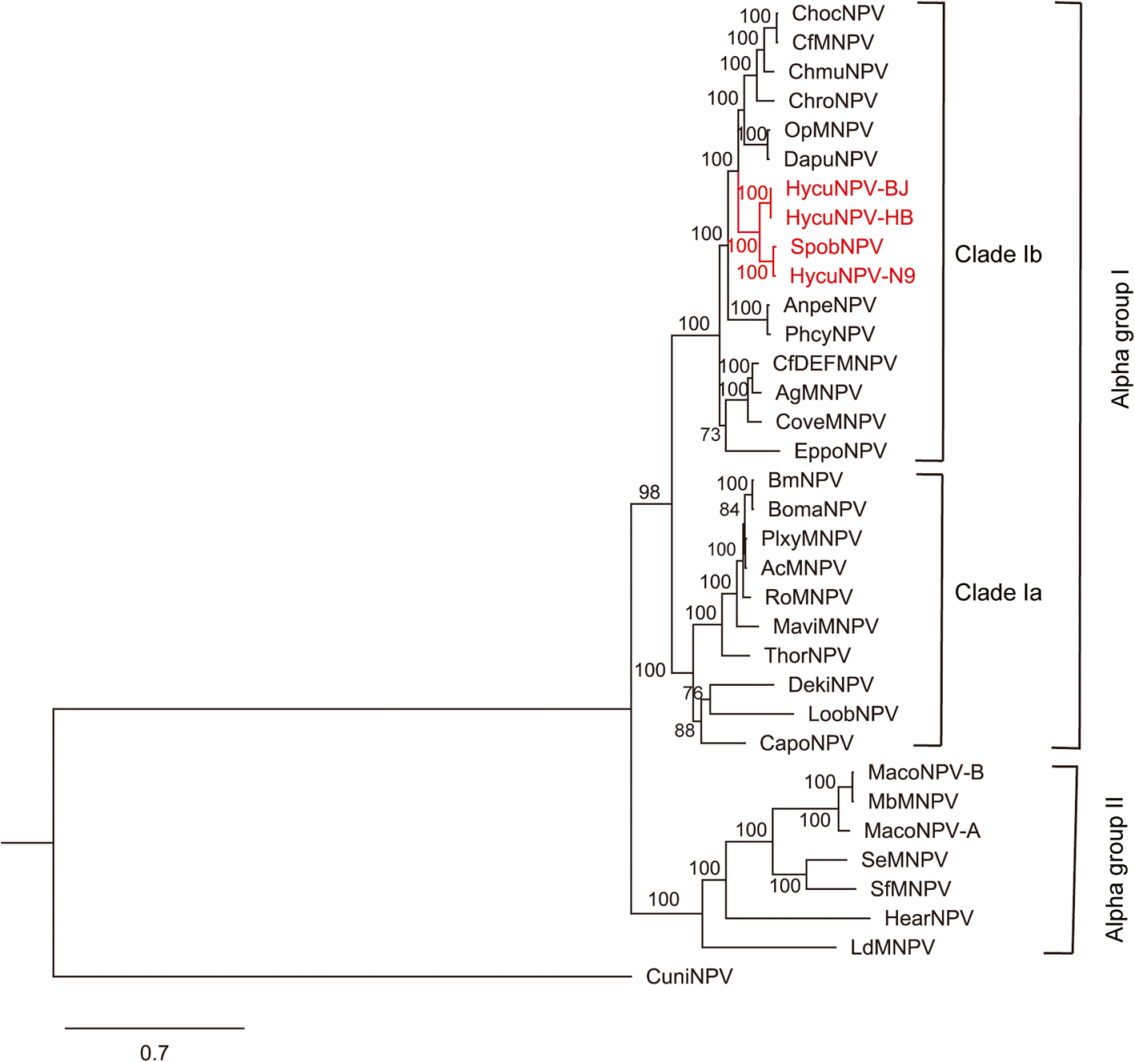
Maximum likelihood tree of HycuNPV isolates and other alphabaculoviruses based on the 38 core genes concatenated amino acid sequences. HycuNPV isolates are highlighted in red, CuniNPV is used as outgroup. Bootstrap values are indicated for each interior branch

The relationships of HycuNPV-HB, HycuNPV-BJ and HycuNPV-N9 with other baculoviruses were inferred using a phylogenetic analysis based on the 38 baculovirus core genes concatenated sequences. The result clearly showed that all the HycuNPV isolates clustered together in Clade Ib *Alphabaculovirus* (Fig. 2)*.* HycuNPV-HB and HycuNPV-BJ formed a subclade and were separated from HycuNPV-N9 and Spilosoma obliqua nucleopolyhedrovirus (SpobNPV). Kimura two-parameter distances (K2P) based on 38 core genes alignment between HycuNPV-BJ and HycuNPV-HB was < 0.021 (Additional file 1: Table S1), while K2P distances between the HycuNPV-N9 and two Chinese isolates were all > 0.072. This indicated that HycuNPV-BJ and HycuNPV-HB belonged to the same species, but they were not same species as the Japanese isolate HycuNPV-N9, they were a novel species of *Alphabaculovirus* that infects *Hyphantria cunea* Drury in China.

### Genome component analysis of HycuNPV isolates

Using de novo assembly, we obtained complete genome sequences of the two Chinese HycuNPV isolates. The genomes of HycuNPV-HB and HycuNPV-BJ were determined to be 132,540 bp (Mean coverage ± SD: 6802 ± 1575) and 130,973 bp (Mean coverage ± SD: 8947 ± 3211) in length, with G + C contents of 47 and 48%, respectively, whereas the HycuNPV-N9 reference genome was 132**,**959 bp, with a G + C content of 46%. When compared with HycuNPV-N9, the HycuNPV-HB and HycuNPV**-**BJ isolates had several gene losses, insertions and rearrangements in their genomes, but they all contained the 38 core genes (Additional file 2: Table S2). The size and number of open reading frames **(**ORFs**)** in HycuNPV**-**HB were similar with those of HycuNPV-BJ. For instance, they both contained *hrf-1* (*host range factor-1*) and lost homologs to the hypothetical genes of ***hycu7***, ***hycu****22*, ***hycu****63*, ***hycu****87*, ***hycu****99* and ***hycu****100*, when compared with HycuNPV-N9**.** Moreover**,** the adjacent ORFs of ***hycu****136* and *odv-e26* were fused together into a single ORF. The HycuNPV-HB and HycuNPV-BJ genomes were annotated and compared with the HycuNPV-N9 genome and other related baculovirus genomes (Additional file 2: Table S2).

**Fig. 3:**
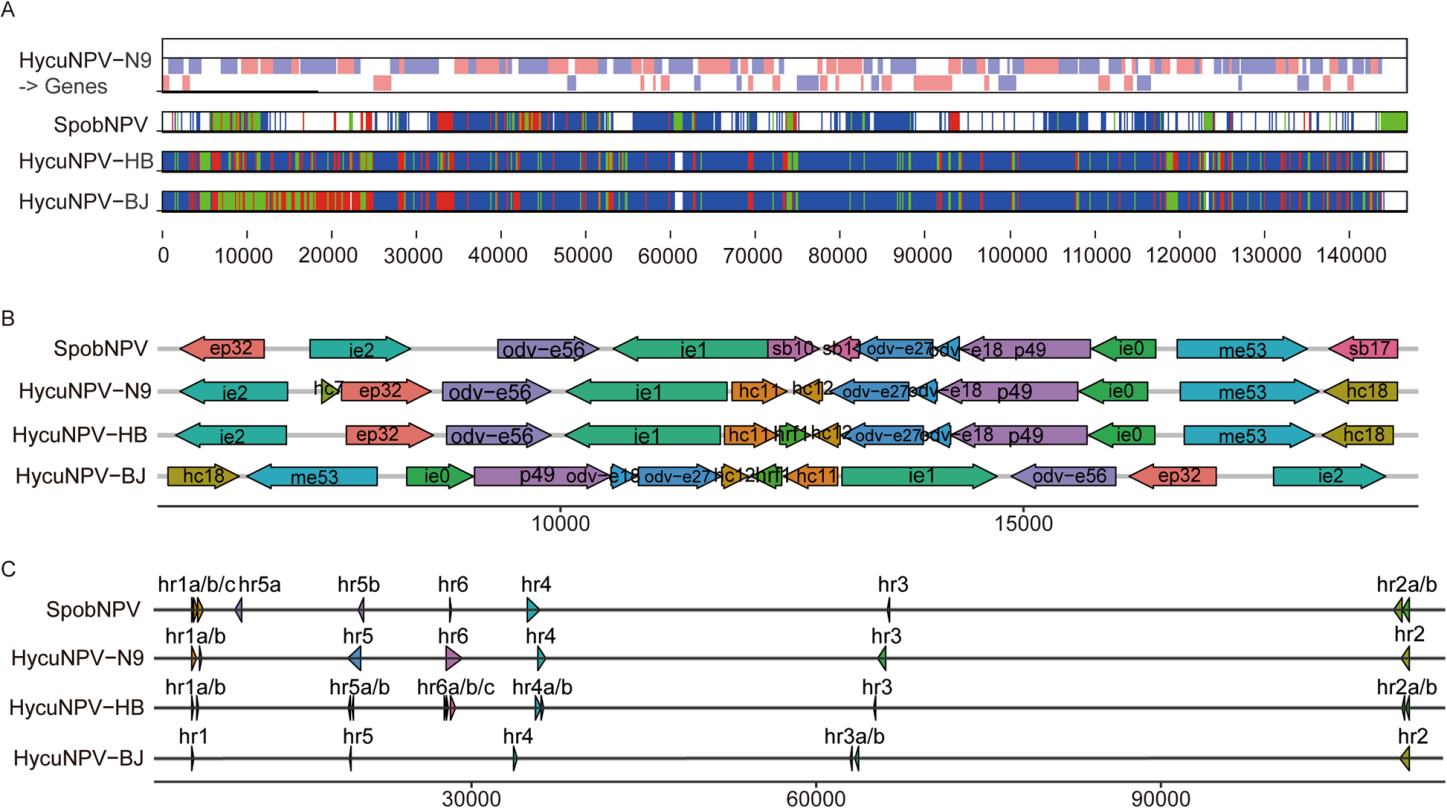
Genomes comparative analysis of HycuNPV isolates based on whole-genomic alignment. **A** Distribution of polymorphic sites in different genomes using Base-by-Base software with the HycuNPV-N9 as reference: blue, nucleotide substitutions; green, insertions; and red, deletions. **B** Comparison of the gene locations in HycuNPV-HB and HycuNPV-BJ genome with those of the corresponding 5–20 kb fragments in HycuNPV-N9 and SpobNPV genome. Arrows denote ORFs and their direction of transcription. **C** The numbers and locations of homologous repeat regions (*hrs*) in each genome

We used Base-by-Base software to perform the whole-genome alignment [11]. The sequence comparison revealed that there were great variations in the genomes of SpobNPV, HycuNPV-HB and HycuNPV-BJ isolates compared with that of HycuNPV-N9. When compared with HycuNPV-N9, the total nucleotide sequence identities of SpobNPV, HycuNPV-HB and HycuNPV-BJ were 96.27, 87.79 and 84.69%, respectively. Variations were detected widely throughout the genomes (more than 10,000 mutations in each Chinese isolate), and were predominantly substitutions, with large insertions and deletions found mainly in homologous repeat regions (*hrs*) (Fig. 3A).

On the basis of a Mauve analysis [12], the gene arrangement of the HycuNPV-HB genome showed higher collinearity with HycuNPV-N9 and SpobNPV than with HycuNPV-BJ, although *ep32* and *ie2* were reversed in SpobNPV genome. In the HycuNPV-BJ genome, two locally collinear block transversions were found in the 5–20 kb genome region, indicating that a gene transversion event had occurred (Additional file 3: Fig. S1). Interestingly, this transversion event was anchored between *hr1* and *hr5*. The *hr1* to *hr5* region spanned approximately 15 kb in the HycuNPV**-**N9 genome and contained 13 genes, *ie2*, *hycu7*, *ep32*, *odv-e56*, *ie1*, *hycu11*, *hycu12*, *odv-e27*, *odv-e18*, *p49*, *ie0*, *me53* and *hycu18* (Fig. 3B).

We also analyzed the distributions and numbers of *hrs* in SpobNPV and all of the HycuNPV genomes. There was an extra *hr7* in SpobNPV genome which was located between *ie2* and *odv-e56*. For all of the HycuNPV genomes, the locations of *hrs* were relatively conserved in the isolates, but the numbers of *hrs* differed slightly, *hr6* was completely lost in the HycuNPV-BJ genome. In addition, most *hrs* (*hr2*, *hr4*, *hr5* and *hr6*) in the HycuNPV-HB genome were split into several segments and scattered along the genome (Fig. 3C).

### Genetic diversity of HycuNPV populations

So far, there were no reports about the complete genome sequences in Chinese HycuNPV isolates, so we used the Japanese isolate HycuNPV-N9 as a reference genome to estimate the diversity of HycuNPV genomes. We combined all the clean reads from two Chinese isolates (919 million for HycuNPV-HB and 1176 million for HycuNPV-BJ, respectively) and mapped them to the HycuNPV-N9 genome (Mean coverage ± SD: 15866 ± 9972). After quality control, we detected 10,393 single nucleotide polymorphisms (SNPs) in the two Chinese isolates (Additional file 4: Table S3)**.** In total, 8599 and 1794 SNPs were found in coding and non-coding regions, respectively (Additional file 5: Fig. S2A). In the coding region, approximately a quarter of the substitutions were non-synonymous mutations, with the remaining being synonymous mutations. Moreover, most of the mutations were found in the third codon position. Among the SNP mutations, including 7415 (71.3%) transitions and 2978 (28.7%) transversions, the transition to transversion ratio was approximately 2.5 (Additional file 5: Fig. S2B). In addition, SNP counts based on the functional group indicating that synonymous were dominant mutations in each functional group, and the total number of SNPs in the non-core genes was higher than the core genes (Additional file 5: Fig. S2C).

**Fig. 4:**
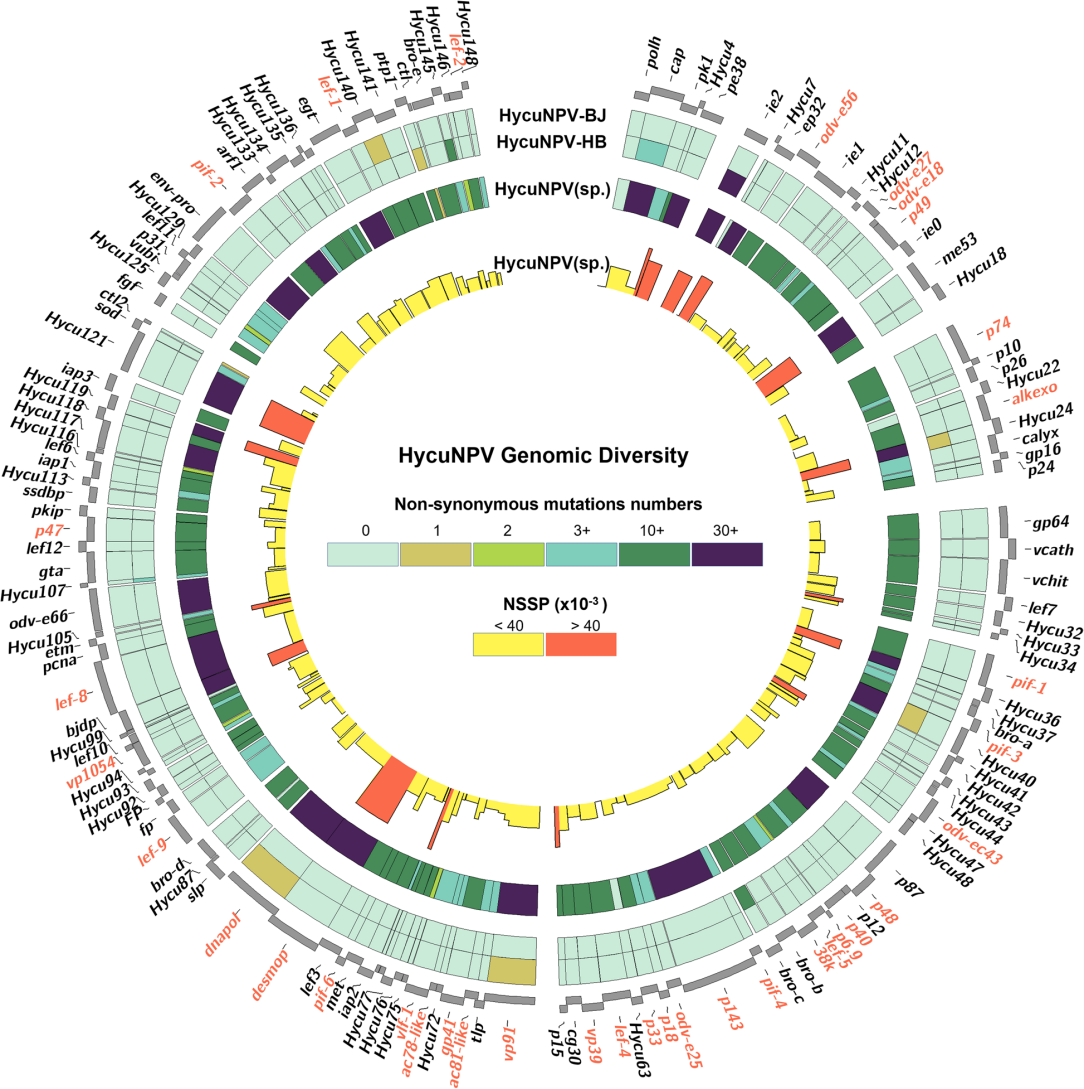
Genetic diversity of the HycuNPV. For each ORF in HycuNPV, the number of nonsynonymous variants was computed by Circos 0.69 (https://github.com/vigsterkr/circos) [13]. Externally of the circular map ORFs are represented in positive and negative sense. Red label genes represent 38 core genes. Each square of the heatmaps on each ring depicts the number of SNPs per gene. The outer rings show HycuNPV-HB and HycuNPV-BJ against its respective representative genome; the middle ring represents SNPs derived from Chinese HycuNPV isolates whole-genome sequence alignment, while the inner ring with colored histogram summarizes the NSSP of Chinese HycuNPV isolates, the height of each histogram indicates the NSSP value for each ORF, the yellow represents the NSSP value less than 40 × 10^− 3^ NSS/bp and the orange represents the NSSP value more than 40 × 10^− 3^ NSS/bp

We analyzed the SNPs distributions in each ORF across the HycuNPV genome (we only considering non-synonymous mutations) (Fig. 4). For the isolate-specific SNPs, since the two Chinese isolates used for sequencing had been purified in vivo cloning, when the clean reads of HycuNPV**-**HB and HycuNPV-BJ were mapped against their own de novo assembly consensus sequence, each isolate showed a very low number of SNPs. For the population-based analysis, we combined all the clean reads of two Chinese isolates and mapped them to the HycuNPV-N9 reference genome, large numbers of SNPs were found in all the ORFs, except that of the *polh* gene, with approximately 19 non-synonymous substitutions per ORF. The genes that showed high levels of diversity were mainly *ie2*, *ep32*, *me53*, *hycu36*, *desmop*, *etm* and *hycu121*, which had non-synonymous substitutions per base pair (NSSP) values of more than 40 × 10^− 3^ NSS/bp (Fig. 4 and Additional file 6: Table S4).

### ORF-associated SNP analysis

**Fig. 5:**
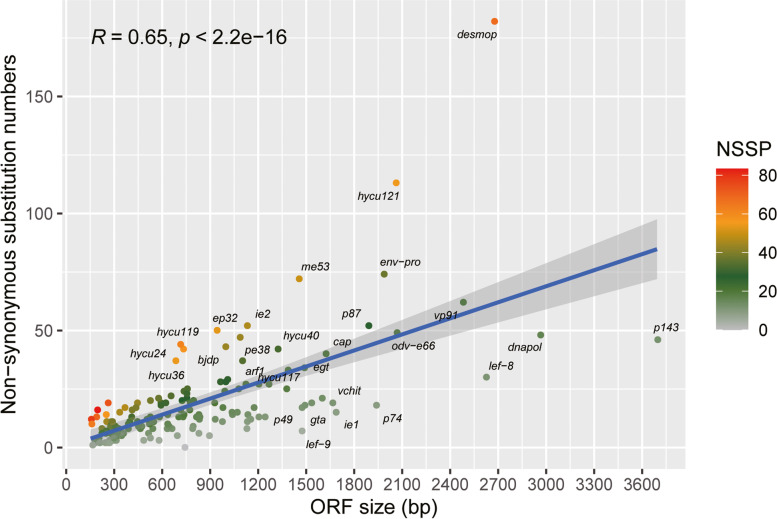
The correlation between non-synonymous substitution numbers and ORF sizes. The correlation coefficient is equal to 0.65, the grey area corresponds to the 95% confidence interval. Each dot represents the non-synonymous substitution numbers for each gene, the color of the dot indicates the NSSP value for each gene (× 10^− 3^ NSS/bp)

In the SNP analyses, we did not use SNPs in the *hr*s and focused on non-synonymous mutations. According to the Brito et al. (2018) method, genetic diversity can be estimated using the values of NSSP. Therefore, the NSSP value for each gene across the genome was calculated. Even though some genes, such as *env-pro* and *p87*, had the greatest numbers of non-synonymous mutations, they were not the most diverse genes and had low levels of diversity (less than 40 × 10^− 3^ NSS/bp) after a standardized treatment using the NSSP method (Fig. 5). The longer the ORF length, the greater the number of non-synonymous mutations. The non-synonymous substitution number correlated with the ORF size (*r* = 0.65, *p* <  0.0001) (Fig. 5). Other genes, such as *hycu78*, *hycu119*, *hycu24* and *hycu121*, showed high levels of diversity (greater than 60 × 10^− 3^ NSS/bp), but with unknown functions. However, most of the genes had low levels of diversity (from 0 to 20 × 10^− 3^ NSS/bp), especially the core genes. However, there was an exception, *desmop*, which was transcribed as a late gene and associated with the production of OBs [14], this gene had a diversity level of 68.09 × 10^− 3^ NSS/bp (Fig. 5 and Additional file 6: Table S4).

### Molecular evolution analysis of Hrf-1

**Fig. 6:**
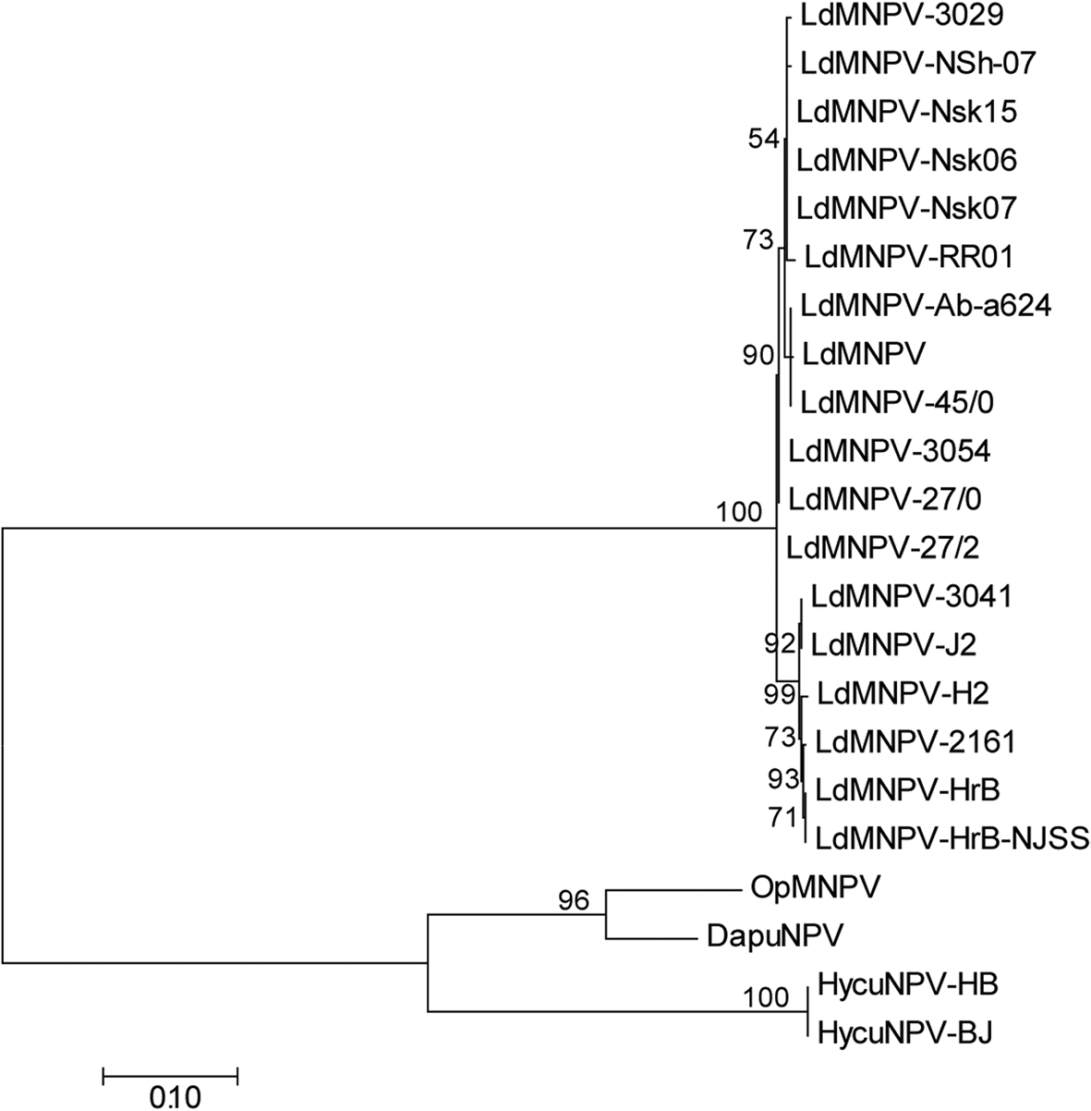
Molecular Evolution analysis of Hrf-1. Phylogenetic analysis is performed using Hrf-1 orthologs, bootstrap values are indicated for each interior branch

A homolog of the Hrf-1 was identified in two Chinese HycuNPV genomes. To understand the evolution of Hrf-1, we performed a BLASTP [15] homology search in NCBI and found that the *hrf-1* gene was only present in baculoviruses. Previous studies suggested *hrf-1* was only present in the genomes of Dasychira pudibunda NPV (DapuNPV), Orgyia pseudotsugata MNPV (OpMNPV), and Lymantria dispar MNPV (LdMNPV) [16]. The phylogenetic analyses of Hrf-1 based on 22 orthologs showed that Hrf-1 were divided into two groups, all of the LdMNPV strains clustered together formed one group, while HycuNPV-HB and HycuNPV-BJ, together with DapuNPV and OpMNPV formed another group (Fig. 6).

### Selective pressure analysis

To further assess whether the HycuNPV population had been subjected to positive or negative selection, we calculated the non-synonymous (dN)/synonymous (dS) substitution rate values for each ORF. Our analysis only identified one gene, *hycu*78, that had a dN/dS values of > 1, which indicated potential positive selection under all of the transition to transversion ratios (TTRs) (Additional file 7: Table S5 and Additional file 8: Fig. S3). The *hycu78* has a small ORF encoding 85 amino acids but with unknown functions. The most homologous protein was Spob075 in SpobNPV, with a 95% amino acid identity, but there was no related functional research. However, *hycu78* had a low similarity with *ac73* (amino acid identity 32%), which is expressed during the late stage of infection and is associated with the production of budded viruses (BVs) and occlusion derived viruses (ODVs) in Autographa californica multiple nucleopolyhedrovirus (AcMNPV) [17]. The vast majority of genes underwent purifying selection, with dN/dS < 1, which indicated that purifying selection was predominant at the whole-genomic level.

### Biological activities of HycuNPV-BJ and HycuNPV-HB isolates

To assess the biological activities of the two Chinese HycuNPV isolates against *H. cunea* larvae in China, droplet-feeding bioassays were performed with third-instar larvae in the Chinese *H. cunea* populations (populations from Japan were not used). In both tests 1 and 2, the median lethal concentration (LC_50_) values of HycuNPV-BJ were approximately 1.8–2-fold higher than those of HycuNPV-HB (Table 1), owing to the higher infectivity of HycuNPV-HB.

**Table 1:** Median lethal concentrations (LC_50_) of HycuNPV isolates against third-instar *H. cunea* larvae

Test	Isolates	LC_50_ (95% CI) (× 10^5^ OBs/mL)	*χ*^*2*^/d.f.	Potency Ratio (95% CI) to HycuNPV-HB^a^
1	HycuNPV-HB	7.517 (1.668, 17.253)	12.383/4	–
	HycuNPV-BJ	15.051 (4.202, 38.738)	13.869/4	2.002 (1.231, 3.258)^b^
2	HycuNPV-HB	7.783 (2.183, 16.918)	11.632/4	–
	HycuNPV-BJ	14.392 (3.470, 35.998)	9.601/4	1.849 (1.063, 3.217)^b^

^a^The potency ratio was calculated by dividing the LC_50_ of HycuNPV-HB by those of the genotypes

^b^indicated that the potency of the genotype was significantly different from that of HycuNPV-HB based on whether the 95% confidence interval of the potency ratio contained 1.0 [18]

When third-instar larvae were inoculated at a 100× LC_50_ concentration, the median survival time (ST_50_) values for the HycuNPV-BJ isolate in two independent tests were significantly longer than those of HycuNPV-HB (*p* <  0.01; Table 2). This result indicated that the killing speed of HycuNPV-HB was faster than that of HycuNPV-BJ**.**

**Table 2:** Median survival times (ST_50_) of the third-instar *H. cunea* larvae inoculated with the HycuNPV isolates

Test	Isolates	*n*	ST_50_ (95% CI) (d.p.i) ^a^	*χ*^*2*^	*p*
1	HycuNPV-HB	48	8.5 (8.085, 8.915)		
	HycuNPV-BJ	44	9.0 (7.555, 10.445)	10.765	0.001
2	HycuNPV-HB	42	7.5 (6.452, 8.548)		
	HycuNPV-BJ	47	9.0 (8.670, 9.330)	18.011	< 0.001

^a^ST_50_ values of insects were determined by the Kaplan–Meier estimator and were reported with 95% CIs. d.p.i.: days post inoculation

### Comparison of the HycuNPV-HB and HycuNPV-BJ genome sequences

Sequence comparisons showed a close to 96% nucleotide sequence identity between the HycuNPV-HB and HycuNPV**-**BJ genomes. The major differences were genome structural variants, including genomic inversions (between *ie2* and *hycu18*), large genomic deletions (*hrs* regions and upstream of the *fgf*, *slp*, *hycu48* and *bro-e* gene) and several gene insertions or deletions. The remaining discrepancies were small numbers of SNPs in homologous genes. Overall, the HycuNPV**-**HB genome was 1567 bp longer than that of HycuNPV-BJ, and the difference was mainly on the *hrs* regions. The numbers of the tandem repeats within *hrs* of the HycuNPV-BJ isolate were obviously less than those of the HycuNPV-HB isolate, especially the complete deletion of *hr6* in HycuNPV-BJ**.** Additionally, *hr1*, *hr2*, *hr4*, *hr5* and *hr6* in the HycuNPV**-**HB genome contained two or more separate repeat regions that were different from each other, whereas the opposite was true for HycuNPV-BJ (Fig. 3C).

The ORF order and content in HycuNPV**-**HB were similar to those of HycuNPV-BJ, with only 10 different genes between them (Additional file 2: Table S2). Seven genes encoding hypothetical proteins with unknown functions were identified near the *hrs* regions, ORF-6, ORF-20, ORF-21, ORF-22 and ORF-70 in HycuNPV-HB, as well as ORF-6 and ORF-7 in HycuNPV-BJ. These protein-encoding genes showed high degrees of homology with each other (amino acid identity **>** 90%), but had no homologs in any reported baculovirus genomes. In addition, ORF-104 was only found in the HycuNPV-BJ genome, and it was homologous with AcMNPV ORF-44 (65.18%) and OpMNPV ORF-49 (63.16%). In addition, they had the same gene inserted between *iap3* and *hycu121,* ORF-119 in the HycuNPV-BJ genome and ORF-121 in the HycuNPV**-**HB genome, respectively, which exhibited relatively low amino acid sequence identities with ***Hyphantria cunea*** granulovirus ORF-55 (32.37%) and Euproctis pseudoconspersa NPV ORF-116 (30%), respectively. ORF-121 in HycuNPV**-**HB had a partial deletion of approximately 300 bp compared with ORF-119 in HycuNPV-BJ.

## Discussion

### General genome characteristics of HycuNPV isolates from China

The *hrf-1* is a host-range gene from Lymantria dispar MNPV (LdMNPV) that prevents translation arrest and promotes AcMNPV replication in non-permissive insects and their cell lines [16]. Here, *hrf-1* was present in the genomes of HycuNPV-HB and HycuNPV-BJ, implying that *hrf-1* contributes to the host range of HycuNPV in China. However, when we inoculated second-instar larvae of *Spodoptera exigua*, *Helicoverpa armigera*, *Spodoptera frugiperda* and *Mythimna separata*, none showed symptoms of viral infection (Additional file 9: Table S6). HycuNPV can infect *Spilosoma oblique*, indicating that the host range of HycuNPV might be limited to family Arctiidae.

In previous studies, *hrf-1* was only found in the genomes of DapuNPV, OpMNPV and LdMNPV [16], but in the present study, we found the *hrf-1* gene in HycuNPV-HB and HycuNPV-BJ genome, while SpobNPV and HycuNPV-N9 does not encode homologues of Hrf-1. As the virus related to the most recent common ancestor of DapuNPV and OpMNPV [10], we inferred that *hrf-1* in HycuNPV-HB and HycuNPV-BJ genomes were acquired in the OpMNPV lineage, and might be lost in HycuNPV-N9 lineage.

The K2P distances proposed by Jehle et al. (2006) based on the *polh/gran*, *lef-8* and *lef-9* were a widely applied method for species demarcation for lepidopteran-specific baculoviruses [19]. Wennmann et al. (2018) had adjusted baculovirus species demarcation criterion based on the 38 core-gene data, the standard thresholds of 0.015 and 0.050 were adjusted to 0.021 and 0.072, respectively, with the K2P demarcation thresholds of 0.021 for the same species and 0.072 for distinct species, and for K2P parameters in-between, more biological information was required [20]. The K2P distances based on 38 core-gene concatenated alignment between two Chinese isolates and HycuNPV-N9 were all > 0.072, moreover, a comparison of the ORF order and content among HycuNPV genomes revealed obvious differences between Chinese and Japanese isolates, with large insertions and deletions, especially the high number of SNPs. Thus, according to the work of Wennmann et al. (2018) [20], it was sufficient to recognize that Chinese isolates and Japanese isolates were different baculovirus species, HycuNPV-BJ and HycuNPV-HB isolates were a novel species of *Alphabaculovirus* that infects *Hyphantria cunea* in China.

HycuNPV-N9 and two Chinese isolates resided on two independent genetic branches, which might be explained by co-evolution with their hosts. *H. cunea* was considered as the native host of HycuNPV. HycuNPV-BJ was isolated from an infected body of Chinese *H. cunea* populations in Beijing in 2013, and HycuNPV-HB was isolated from the local populations in Hubei Province. In Asia, *H. cunea* spread from Japan to South Korea, and then to China. Ozaki revealed that the variations in *H. cunea* in Japan was the result of an evolutionary adaptive response to the climate in different regions of Japan acting through natural selection [21]. In addition, a population genetic study in 2016 showed that *H. cunea* populations in China fall into two distinctive genetic groups [3]. Thus, it might be hypothesized that the dramatic level of host intraspecific variation would likely lead to genetic variations in HycuNPV genome that were tightly adapted to the Chinese *H. cunea* populations in order to facilitate viral-reproductive success.

In the HycuNPV-BJ genome, there was a gene transversion event occurred between *hr1* and *hr5* which spanned approximately 15 kb in the HycuNPV**-**N9 genome. According to previous genome evidence, *hrs* were more prone to sequence variation than other genome regions, because they were usually found in more than one copy [22]. It has been reported that some genes in SfMNPV-ColA genome were acquired by a recent homologous recombination process between *Spodoptera frugiperda* and *Spodoptera litura* [23]. DNA crossover may occur between two loci from one genome, between genotype variants of the same species, or between genomes from different virus species co-infecting the same host [24]. As *hrs* were recognized as a recombination facilitators, we inferred that the gene transversion in HycuNPV-BJ genome was the result of homologous recombination between *hr1* and *hr5* to maintaining adaptability in nature.

### Genetic diversity in HycuNPV genomes

Several works about the genetic diversity in baculovirus had been reported by using a deep sequencing approach. Fan et al. (2021) analyzed the distribution of SNPs in twenty isolates of Cydia pomonella granulovirus using the data sets of re-sequenced genomes with mean read depth ranged from 540 to 4345 [25], Masson et al. (2021) studied the genomic diversity of SfMNPV ARG-M genome with an average coverage of 52,313 and standard deviation of 4052 [26]. In our present study, we used a resequencing approach using HycuNPV-N9 as a reference, resulting in high level of coverage (resequencing mean coverage ± SD: 15866 ± 9972) to explore the diversity in HycuNPV genomes. For intra-populational genetic variants, we found limited non-synonymous substitutions within each Chinese isolate, this may be due to the single and highly pure genotype of the two Chinese isolates. There are very limited within-isolate polymorphisms in each Pakistani *Spodoptera litura* NPV [27], and a similar situations has been reported in *Spodoptera frugiperda* MNPV [28] and AcMNPV [29]. For inter-isolate non-synonymous mutations, the most variable genes were *hycu78*, *desmop*, *p15*, *hycu36*, *hycu118*, *etm*, *me53* and *ep32*, having genetic diversity levels greater than 40 × 10^− 3^ NSS/bp, whereas most of the genes had values lower than the average diversity of the whole genome (23.28 × 10^− 3^ NSS/bp). The average diversity found in HycuNPV was higher than previous reports for *Spodoptera frugiperda* MNPV (5.3 × 10^− 3^ NSS/bp) [26] and ErelGV (7.0 × 10^− 3^ NSS/bp) [30].

By grouping HycuNPV genes on the basis of their main function, the identified SNPs were classified into auxiliary, replication, transcription, structural, oral infection and unknown function groups. The genes with the highest levels of diversity identified in our study were in the unknown function group, such as *hycu78*, *hycu4*, *hycu24*, *hycu119* and *ep32* (Additional file 10: Fig. S4). Mutations were found in all gene functional classes, but there were no significant differences among functional groups, and there were also no correlations between high-frequency mutations and functional groups, similar phenomena have also observed in ErelGV isolates [30], which indicated that the variations are random and irregular in the viral genome. Baculovirus early genes encode all the proteins needed for viral DNA replication. In this study, most of the early genes, such as *ep32*, *etm*, *p15*, *ie2*, *pe38* and *hycu78*, showed higher levels of diversity than late genes, but only 5% of late genes had high NSSP values (such as *desmop*, *hycu24 and bjdp*), indicating that early genes exhibited significant higher diversity levels than late genes (Additional file 10: Fig. S4).

Thézé et al. (2014) reported the genomic diversity in European SeMNPV isolates, and pointed that the differences in baculovirus virulence and transmission phenotypes involve multiple molecular pathways, the high genomic variation found in all gene functional classes could result in functional or phenotypic characteristics changes, such as pathogenicity, speed of kill and yield [31]. According to this theory, it is likely that the high levels of genetic diversity observed in viral early and late genes in HycuNPV genome may be involved in establishing viral infections and maintaining a persistent infection in the host cells, because genetic remodeling is a main driver of adaptation during virus–host coevolutionary processes.

### The *hrs* are the main sources of differences in virulence between HB and BJ isolates

In bioassays, the HycuNPV**-**HB isolate was two folds more pathogenic than the HycuNPV-BJ against *H. cunea* larvae. Moreover, HycuNPV**-**HB killed the third-instar larvae of *H. cunea* significantly faster than HycuNPV-BJ (*p* ≤ 0.001). The virulence of a virus is potentially associated with the genetic composition [32]. The major differences between two genomes were structural variants, especially the large genomic deletions in *hrs* regions and upstream of the *fgf*, *slp* (*gp37*), *hycu48* and *bro-e* genes in the HycuNPV-BJ genome. Fgf is a secreted protein that plays a role in the dissemination of a viral infection from the midgut of the insect hosts, and a deletion of the *fgf* in AcMNPV results in a delayed time of death for larvae [33]. Gp37 is associated with the envelopes of BV and ODV. In baculovirus genomes, the promoter is generally located in a region 200-bp upstream of a gene. Deleting the upstream region affects the integrity of the promoter and may reduce the transcriptional efficiency of the gene. Therefore, the promoter sequence likely influences the speed of killing and potency of the HycuNPV-BJ genome.

The *hrs* can serve as origins of DNA replication and as enhancers of gene expression [34]. Furthermore, their numbers correlate with replication efficiency [35]. Gene transcription may be related to the gene’s distance from the *hrs* [36]. Moreover, in AcMNPV, *ac-hr1*, *ac-hr2* and *ac-hr3* enhance the expression of their surrounding genes, as well as gene transcription within a surrounding region of approximately 10–30 kb [37]. Like most baculoviruses, HycuNPV also contains multiple copies of *hrs* distributed throughout the genome, and there were less palindromic sequences in the *hrs* of HycuNPV-BJ than in HycuNPV-HB, including a complete deletion of *hr6* in HycuNPV-BJ. This is the largest *hr* in the HycuNPV genome and is closely related to *ac-hr5* at the nucleotide sequence level. The *hycu-hr6* is a powerful and versatile promoter enhancer in insect expression systems, and it enhances the activities of distinct baculovirus *ie* promoters, as well as the activity of the cellular *hsp70* promoter in lepidopteran and dipteran cell lines [38]. Moreover, in the HycuNPV genome, *gp64* is located downstream of *hr6*, GP64 is an envelope fusion protein that is involved in initiating the infection of other cells. Overall, it is possible that the differences in the *hr* regions are key factors that affect the virulence of HycuNPV isolates. Of course, this conclusion is needed to be further confirmed. According to previous research in AcMNPV [37], we can use a synthetic platform to investigate the function of *hrs* in HycuNPV genome. By constructing the synthesized HycuNPV genomes with no *hrs* or with only a single *hr* via TAR in yeast, we will rescue viruses by transfecting the synthetic genomes into insect host cells and characterized the growth and transcription features of synthetic viruses. This is a systematic job, but the functional studies about *hrs* will be helpful to us for understanding the phenotypic effect of the genomic variations and enriching our knowledge for *hrs*.

## Conclusions

We first report a genome-wide study of Chinese HycuNPV isolates and described their genetic diversity at the whole-genome level, our results enrich the current understanding of this species in ICTV. We reveals HycuNPV-BJ and HycuNPV-HB were a novel species of *Alphabaculovirus* that infects *Hyphantria cunea* Drury in China, the divergence of the phylogenetic tree and gene contents indicated significant differences in the HycuNPV genomes of Chinese and Japanese isolates. Because of its the high activity level and rapid killing of *H. cunea* larvae**,** HycuNPV**-**HB is likely better suited to be developed as a biological insecticide for the control of ***H. cunea*** larval populations in China.

## Methods

### Insects and virus isolation

A population of *H. cunea* larvae was collected from Sanlicheng Town (31°43′N, 114°8′E) in Dawu City, Hubei Province, China, in 2019. These *H. cunea* larvae were reared on an artificial diet for pass generations and maintained in an acclimatized room at 25 ± 1 °C with 50% ± 5% relative humidity and a 16:8 (light/dark) photoperiod [9]. HycuNPV-HB was isolated from a *H. cunea* population showing characteristics of baculovirus infection in the laboratory. HycuNPV-BJ was isolated from the infected body of an *H. cunea* larva from Beijing (40°01′N, 116°25′E) in 2013. The two isolates were propagated in third-instar *H. cunea* larvae. The OBs were purified in accordance with a previous protocol [39].

### SEM and TEM

For SEM, purified OBs were spread onto a piece of foil paper, dried naturally overnight, sputter-coated with gold and observed using a scanning electron microscope (SU8010, Hitachi, Tokyo, Japan) at an accelerating voltage of 10 kV. For TEM, the purified OBs were resin-embedded, sectioned and stained with lead citrate for 30 min as described previously [18]. Stained ultrathin sections were examined using a transmission electron microscope (H-800, Hitachi, Tokyo, Japan) at an accelerating voltage of 200 kV.

### Bioassay

The insecticidal activity assays were only done with the Chinese *H. cunea* populations (populations from Japan were not used), we used the droplet-feeding method as described by Lei [40]. For HycuNPV-HB and HycuNPV-BJ isolates, OB suspensions were diluted into six concentrations (3 ×  10^5^, 9 × 10^5^, 3 × 10^6^, 9 × 10^6^, 3 × 10^7^ and 9 × 10^7^ OBs/mL). In total, 50 third-instar larvae from the Beijing *H. cunea* population were tested per concentration in each assay. After a 24-h starvation treatment, each larva was fed 1 g of artificial diet containing 200 μL of viral suspension, and control larvae were fed the same quantity of artificial diet treated with distilled water. The larvae were incubated under the same conditions as the healthy larvae. From 3 days after infection, mortality was recorded daily until all the larvae had either pupated or died. All the bioassays were conducted in duplicate. The LC_50_ values and potency ratios of the genotypes to the field isolates were calculated using our previously published R (version 3.6.3) scripts [41], which corrected for natural mortality in accordance with Abbott’s formula.

To determine the killing speed of the HycuNPV variants, third-instar larvae were fed OBs at 100× LC_50_, using the droplet-feeding method. The larval mortality was checked approximately every day after the initial 3-day post-inoculation period, recorded twice daily from 6 to 10 days, and every day until all the larvae had either pupated or died. Bioassays were conducted in duplicate The ST_50_ values and their 95% confidence intervals were calculated using Kaplan–Meier curves with the ‘survival’ package in R (version 3.6.3) [42]. Differences between survival curves among the test larvae dosed with the HycuNPV isolates were compared using the log-rank test [43].

The inoculation study was carried out to confirm the cross infectivity of the virus, for HycuNPV-HB and HycuNPV-BJ isolates, the concentration of OB suspensions were both 1 × 10^8^ OBs/mL, and then inoculated 24 second-instar larvae of *Spodoptera exigua*, *Helicoverpa armigera*, *Spodoptera frugiperda* and *Mythimna separata*, respectively, the larvae were incubated under the same conditions, mortality was recorded follow the method described above. All the bioassays were conducted in duplicate.

### Genomic sequencing, assembly and annotation analysis

For each virus isolate to be sequenced, DNA was extracted from OB suspensions using the standard protocol as previously described [44]. An aliquot containing 5 μg of DNA for each virus was used to construct a paired-end library, which was sequenced using Illumina HiSeq PE150 at Beijing Novogene Bioinformatics Technology Co., Ltd. (Beijing, China), generating 1078 and 1520 million paired reads for HycuNPV-HB and HycuNPV-BJ, respectively. All the sequenced reads were filtered to remove the low quality and short reads to produce the final clean reads. The de novo assembly method was used with HycuNPV-N9 (Accession number: NC_007767.1) as a reference genome. The clean data (919 million for HycuNPV-HB and 1176 million for HycuNPV-BJ, respectively) were assembled de novo using Geneious Prime 2021.2.2 (https://www.geneious.com) with default parameters. Gaps and ambiguities between contigs were resolved by PCR amplifying the corresponding sequence regions from viral DNA and sequencing the PCR products. The ORFs were predicted using Geneious Prime and corrected by comparison with the HycuNPV-N9 genome annotation. They were used as query in a BLAST search against the Ref Seq protein database from all organisms deposited in GenBank (https://blast.ncbi.nlm.nih.gov/Blast.cgi). ORFs that encoded at least 50 amino acids and had a minimal overlap with the neighboring ORFs were selected for further characterization [45]. We set the *polyhedrin* start codon as the genome start position. The *hrs* were predicted using the online tool Tandem Repeats Finder (https://www.ncbi.nlm.nih.gov/orffinder/) [46]. The complete genome sequences and annotations for HycuNPV-HB and HycuNPV-BJ were deposited in GenBank under accession numbers OL686893 and OL686894, respectively.

For the genome collinearity analysis, all the annotated ORFs and repeat regions were compared with the genome sequence of HycuNPV-N9 using the Mauve algorithm implemented in Geneious Prime software [12]. The distribution of polymorphic sites in the HycuNPV whole-genome alignment was inferred using the Base-By-Base 3.0 software [11].

### Phylogenetic analysis

The de novo assembly sequences for the HycuNPV-HB and HycuNPV-BJ genomes, as well as that of HycuNPV-N9 and other 31 publicly available baculovirus genomes from the NCBI database were used to construct a phylogenetic tree (Additional file 11: Table S7), and CuniNPV was chosen as the outgroup. The 38 core genes concatenated amino acid sequence alignment was constructed using MAFFT [47]. A maximum-likelihood tree was inferred using PhyML 3.1 [48] with the substitution model LG + G + I. The reliability of the tree was explored using a bootstrap analysis with 1000 replicates [49]. To verify whether the virus corresponds to a new species, the Kimura two-parameter distances (K-2-P) were calculated for the 38 core genes concatenated nucleotide sequences by using the pairwise distance calculation of MEGA 7 applying the Kimura 2-parameter model [19, 20]. BLASTP [15] homology search was used to identify orthologs of Hrf-1 present in all of the baculovirus and other species in NCBI non-redundant protein databases (Additional file 11: Table S7), 22 protein sequences were retrieved and used to aligned using ClustalW [50]. A phylogenetic tree was reconstructed using the Maximum Likelihood method based on the Jones–Thornton–Taylor (JTT) model with 1000 bootstrap values using MEGA7 software [51].

### Genetic diversity of HycuNPV

To explore the diversity of the HycuNPV populations in China, we combined all the clean reads of the HycuNPV-HB and HycuNPV-BJ isolates and mapped them to the HycuNPV-N9 reference genome (Mean coverage ± SD: 15866 ± 9972) using Geneious Prime with default parameters for the population-based analysis. The SNPs were detected using the Find Variations/SNPs plug-in with the following criteria: low-frequency variants > 0.05 and read depth > 100 [25]. To ensure the reliability of our SNPs, we also used Geneious Prime to visually validate variations to avoid potential false-positive sites. To evaluate the intra-populational diversity, each sequence dataset of the Chinese isolates was mapped against its respective representative genome for the individual-based analysis, and the SNPs were identified using the above method. Based on the gene-function classifications of AcMNPV and other related baculoviruses [45, 52, 53], all the SNPs identified in ORFs were grouped into the following six functional groups: auxiliary, replication, transcriptional, oral infection, structural and unknown function. The genetic diversity was estimated using NSSP (non-synonymous substitutions per base pair) values [30], and the result was plotted using the R statistical programming language. The SNP distribution’s circular map was constructed using Circos 0.69 (https://github.com/vigsterkr/circos) [13].

### Analysis of selective pressures

According to previous literature [54], selective pressure is generally assumed to be defined by the *ω* value or the dN/dS ratio. If dN/dS > 1, then the gene is under strong positive selection. If dN/dS < 1, then the gene is undergoing negative or purifying selection, and if dN/dS = 1, then neutral evolution has occurred [55]. To perform the dN/dS analysis on each gene, we used a standard method described previously [27]. We first generated an expectation for the number of possible synonymous mutations and non-synonymous mutations in each ORF using the HycuNPV-N9 genome as a reference sequence. Then, we assumed TTR values of 1, 3 and 5, independently, and generated predictions for each value. Custom R scripts were used to generate these predictions. Finally, using the synonymous and non-synonymous substitutions observed in each ORF, together with the generated expectations, we calculated the dN/dS value for each ORF. The R code is available in the supplementary information (Additional file 12: R code).

## Supplementary Information

**Additional file 1.**

**Additional file 2.**

**Additional file 3.**

**Additional file 4.**

**Additional file 5.**

**Additional file 6.**

**Additional file 7.**

**Additional file 8.**

**Additional file 9.**

**Additional file 10.**

**Additional file 11.**

**Additional file 12.**

## Data Availability

The genomes sequenced in this work are available in GenBank under the accession numbers: OL686893 (https://www.ncbi.nlm.nih.gov/nuccore/OL686893) and OL686894 (https://www.ncbi.nlm.nih.gov/nuccore/OL686894).

## Abbreviations

NPV: Nucleopolydedrovirus
MNPV: Multiple nucleopolydedrovirus
ORF: Open reading frame
*hrs*: Homologous repeat regions
*hrf-1*: Host range factor 1
HycuNPV: *Hyphantria cunea* nucleopolyhedrovirus
SpobNPV: Spilosoma obliqua nucleopolyhedrovirus
AcMNPV: Autographa californica multiple nucleopolyhedrovirus
LdMNPV: Lymantria dispar MNPV
DapuNPV: Dasychira pudibunda NPV
OpMNPV: Orgyia pseudotsugata MNPV
ErelGV: Erinnyis ello granulovirus
SEM: Scanning electron microscopy
TEM: Transmission electron microscopy
SNP: Single nucleotide polymorphism
NSSP: The number of non-synonymous substitutions per base
dN/dS: Non-synonymous/synonymous substitution rate ratio
OBs: Occlusion bodies
BV: Budded virus
ODV: Occlusion-derived virus
LC_50_: Median lethal concentration
ST_50_: Median survival time
TTR: Transition to transversion ratio
