# Supplementary material for: Genomic analysis of two Chinese isolates of hyphantria cunea nucleopolyhedrovirus reveals a novel species of alphabaculovirus that infects hyphantria cunea drury (lepidoptera: arctiidae)
Source: BMC Genomics. 2022 May 13;23:367. doi: 10.1186/s12864-022-08604-7 (PMC9107115; doi:10.1186/s12864-022-08604-7)

Fig. S1 Genome collinearity analysis between HycuNPV isolates and other baculovirus genomes using Mauve software. Each colored block is genetically similar, with the profile height inside each block showing the similarity percentages. A block above the center line indicates that the aligned region is in the forward orientation relative to the first genome, a block below the center line indicates regions that align in the reverse-complement (inverse) orientation. Using HycuNPV-N9 as the reference, two blocks were inverted between the HycuNPV-HB and HycuNPV-BJ genomes.

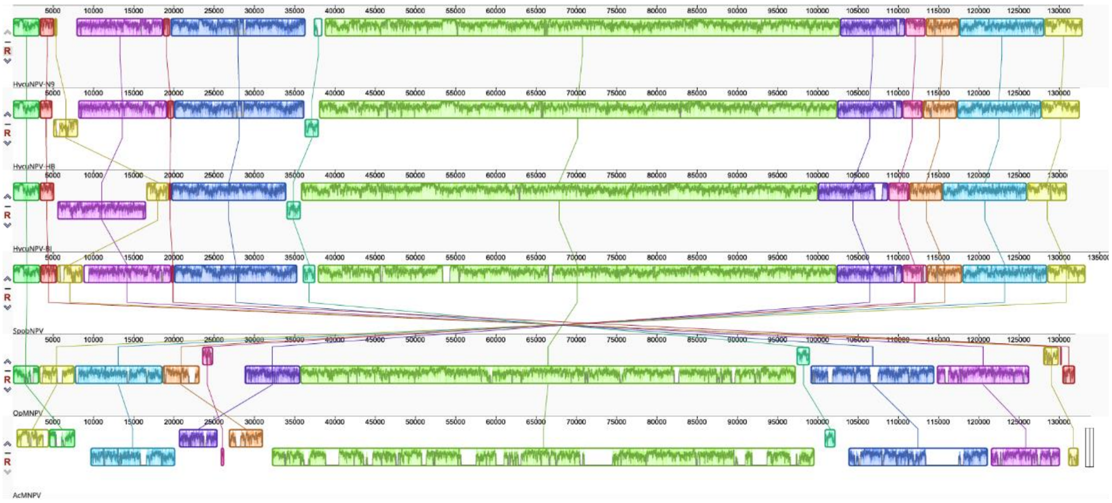

Supplement: Supplementary file 3 — Additional file 3. [file 12864_2022_8604_MOESM3_ESM.pdf]
