# Supplementary material for: Genomic analysis of two Chinese isolates of hyphantria cunea nucleopolyhedrovirus reveals a novel species of alphabaculovirus that infects hyphantria cunea drury (lepidoptera: arctiidae)
Source: BMC Genomics. 2022 May 13;23:367. doi: 10.1186/s12864-022-08604-7 (PMC9107115; doi:10.1186/s12864-022-08604-7)

Fig. S2 Overview of SNPs identified in two Chinese HycuNPV genomes. (A) SNP counts based on their exchange characteristics (transition or transversion), their genome position (coding or non-coding), their coding influence (synonymous or non-synonymous) and their codon position (first, second or third). (B) SNP counts based on their SNP class. (C) SNP counts based on their functional group.

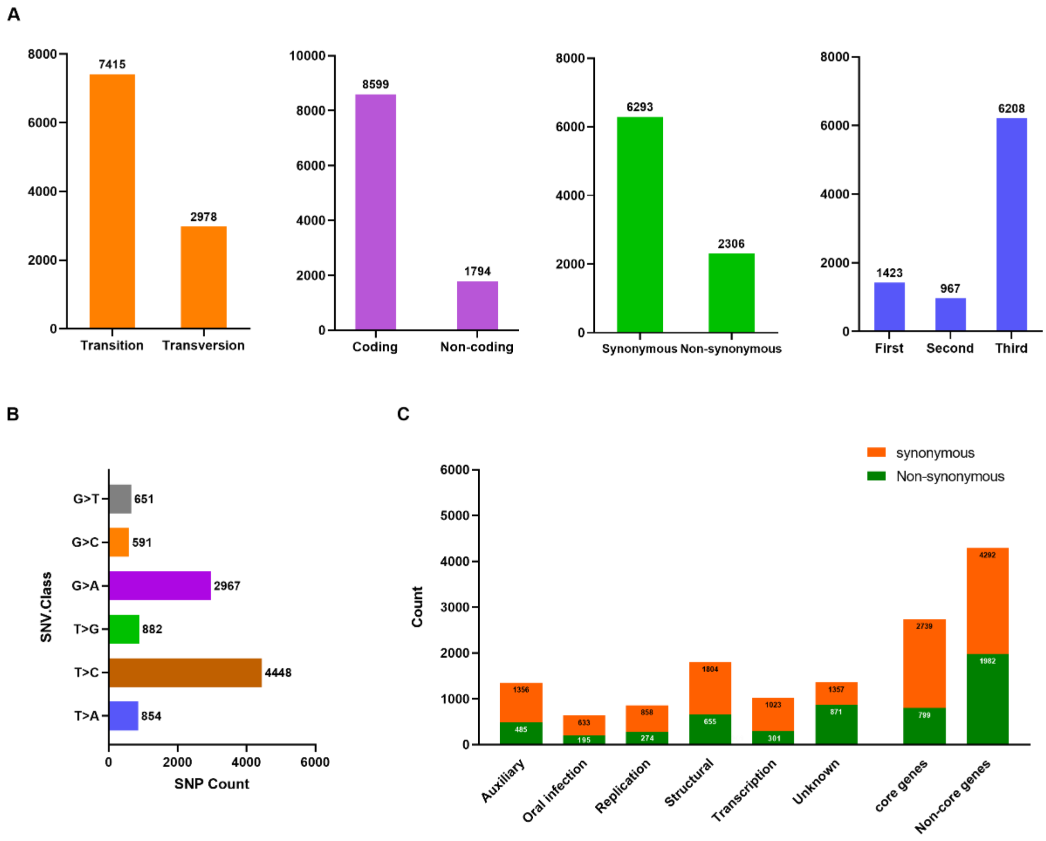

Supplement: Supplementary file 5 — Additional file 5. [file 12864_2022_8604_MOESM5_ESM.pdf]
