# Supplementary material for: Genomic analysis of two Chinese isolates of hyphantria cunea nucleopolyhedrovirus reveals a novel species of alphabaculovirus that infects hyphantria cunea drury (lepidoptera: arctiidae)
Source: BMC Genomics. 2022 May 13;23:367. doi: 10.1186/s12864-022-08604-7 (PMC9107115; doi:10.1186/s12864-022-08604-7)

Fig. S3 The dN/dS analysis results. The *x*-axis indicates ORF numbers corresponding to the HycuNPV-N9 reference genome. dN/dS values assuming different mutational biases are presented as follows: TTR values of 1 (circles), 3 (triangles) and 5 (squares). The black line represents dN/dS = 1, which indicates neutral evolution. When dN/dS < 1, then there is purifying selection, whereas when dN/dS > 1, then there is positive selection.

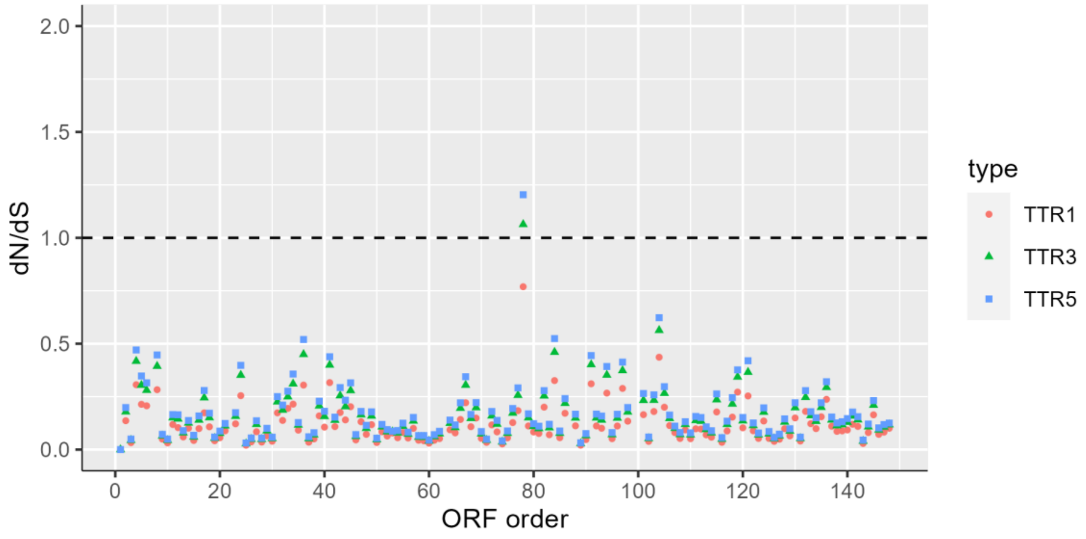

Supplement: Supplementary file 8 — Additional file 8. [file 12864_2022_8604_MOESM8_ESM.pdf]
