# Supplementary material for: Genomic analysis of two Chinese isolates of hyphantria cunea nucleopolyhedrovirus reveals a novel species of alphabaculovirus that infects hyphantria cunea drury (lepidoptera: arctiidae)
Source: BMC Genomics. 2022 May 13;23:367. doi: 10.1186/s12864-022-08604-7 (PMC9107115; doi:10.1186/s12864-022-08604-7)
Supplement: Supplementary file 9 — Additional file 9. [file 12864_2022_8604_MOESM9_ESM.pdf]

Table S6. Feeding experiments with the two Chinese HycuNPV isolates in second-instar larvae of *Helicoverpa armigera*, *Spodoptera frugiperda*, *Spodoptera exigua* and *Mythimna separata* by *per os*.

**Insect**	**HycuNPV-HB**			**HycuNPV-BJ**		
	treat (n)	dead (n)	mortality (%)	treat (n)	dead (n)	mortality (%)
**Test 1**						
Helicoverpa armigera	24	0	0	24	0	0
Spodoptera frugiperda	24	0	0	24	0	0
Spodoptera exigua	24	0	0	24	0	0
Mythimna separata	24	0	0	24	0	0
**Test 2**						
Helicoverpa armigera	24	0	0	24	0	0
Spodoptera frugiperda	24	0	0	24	0	0
Spodoptera exigua	24	0	0	24	0	0
Mythimna separata	24	0	0	24	0	0
