# Supplementary material for: Genomic analysis of two Chinese isolates of hyphantria cunea nucleopolyhedrovirus reveals a novel species of alphabaculovirus that infects hyphantria cunea drury (lepidoptera: arctiidae)
Source: BMC Genomics. 2022 May 13;23:367. doi: 10.1186/s12864-022-08604-7 (PMC9107115; doi:10.1186/s12864-022-08604-7)

Fig. S4 SNP analysis based on functional categories. (A) Genetic diversity in ORFs based on functional groups. (B) The average NSSP levels of each functional groups. (C) The levels of diversity between non-core and core genes among different functional groups. (D) The levels of diversity between early and late genes.

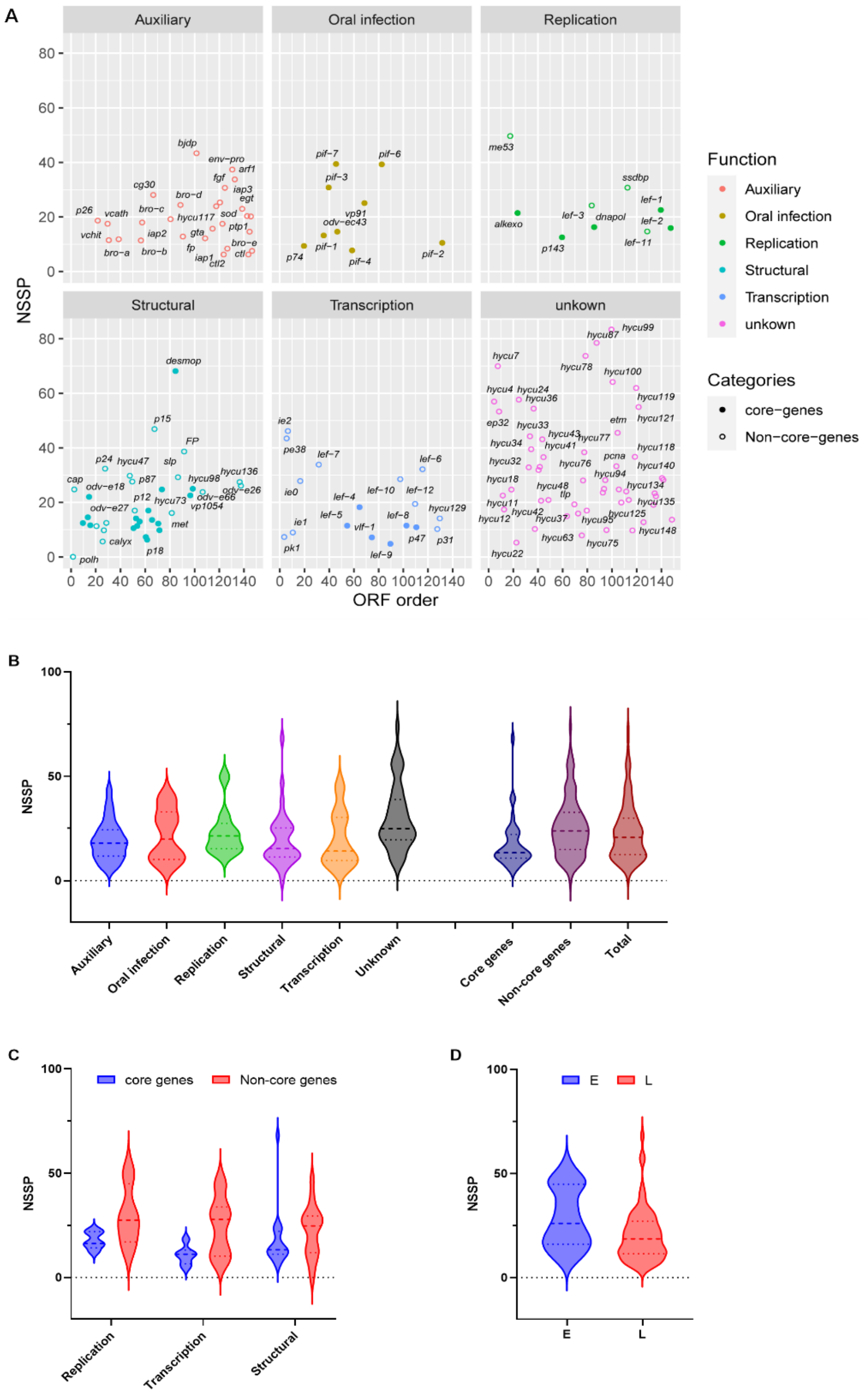

Supplement: Supplementary file 10 — Additional file 10. [file 12864_2022_8604_MOESM10_ESM.pdf]
